# Exploring Factors Associated with Limited Male Partner Involvement in Maternal Health: A Sesotho Socio-Cultural Perspective from the Free State, South Africa

**DOI:** 10.3390/ijerph21111482

**Published:** 2024-11-07

**Authors:** Michelle Engelbrecht, Ngwi Mulu, Gladys Kigozi-Male

**Affiliations:** Centre for Health Systems Research & Development, Faculty of the Humanities, University of the Free State, P.O. Box 339, Bloemfontein 9300, South Africa; ngwi.mulu@gmail.com (N.M.); kigozign@ufs.ac.za (G.K.-M.)

**Keywords:** maternal health, male partner involvement, antenatal care, delivery, barriers

## Abstract

Despite efforts to promote gender-inclusive approaches to maternal health, male partner involvement (MPI) remains limited, underscoring the need for a comprehensive understanding of the factors associated with a lack of MPI. A mixed method, concurrent/parallel design was applied to explore MPI in maternal health and to identify factors associated with limited MPI. Data collection entailed a cross-sectional survey among 407 fathers together with 16 focus group discussions with men and women of reproductive age and eight community discussions with community leaders. MPI was defined in terms of communication, decision making, physical and emotional support and physical presence at ANC. Data was subjected to descriptive, multiple linear regression and thematic analysis. Three out of five participants (63.9%) scored above the mean for higher levels of MPI in maternal health. Factors significantly associated with a lower level of MPI were younger age, unemployment, men not living with the mother of their youngest child, men who had not had a male role model involved in domestic chores, and men who were not interested in attending future maternal health programmes. In addition, the qualitative analysis also identified relationship problems, family conflicts, health systems barriers and alcohol abuse as hinderances to MPI in maternal health. Health systems capacity is required for the promotion of male partner interventions that are in line with socio-cultural practices and gender norms.

## 1. Introduction

The importance of male partner involvement (MPI) in maternal health care, i.e., the health of women during pregnancy, childbirth and the postnatal period [1], became increasingly prominent in global health discourse following the ICDP Programme of Action, adopted in the 1990s, which called for men and women to be equal partners, and for men to take responsibility for their sexual and reproductive behaviour [2]. More recently, the World Health Organization (WHO) recommended that maternal and neonatal health outcomes could be improved through the active involvement of men during pregnancy, delivery and the postpartum period [3], with research supporting this recommendation [4,5,6]. However, the WHO also cautioned that such interventions should respect women’s autonomy in decision making regarding their health and that of their newborns [3].

What is MPI In maternal health? According to Galle et al. [7], no standard definition or measure exists for MPI in maternal health. In response, the authors developed a male involvement framework to assess MPI in maternal health. From their systematic review of research conducted since 2000—the majority of which (43%) were conducted in Africa—they identified five overarching categories of involvement related to: (1) communication; (2) decision making; (3) practical support; (4) physical support; and (5) emotional support. A scoping review of enablers and barriers to men’s involvement in maternal health in sub-Saharan Africa [8] also defined male involvement in terms of Galle et al.’s [7] framework. A qualitative study exploring the meaning and understanding of MPI during pregnancy among men in a rural area of Mpumalanga Province, South Africa, revealed that MPI encompassed support for women (i.e., financial support, assisting with physical tasks and emotional support) as well as accompanying partners to the clinic, and keeping a place for her in the queue [9]. Still in Mpumalanga, another study focused on MPI in the prevention of mother-to-child transmission of HIV, used a series of eight questions focused on attending ANC visits, discussing ANC and financial support, to measure MPI [10].

Despite efforts to promote gender-inclusive approaches to maternal health, men’s participation remains limited in many regions worldwide [11], particularly in sub-Saharan Africa where research identified a lack of involvement in antenatal care (ANC)—38.2% in Ethiopia [12], 23.6% in Ghana [13], 29.2% in Nigeria [14], 33% in South Africa [15], delivery—52.3% in Ghana [13], 1% in South Africa [15], and postnatal care—31.8% in Ghana [13], 31.6% in Nigeria [14], underscoring the need for a comprehensive understanding of the factors associated with a lack of MPI in maternal health care.

Reasons for the lack of MPI in maternal health have been identified and classified as cultural beliefs and practices [6,8,11,14,16,17,18,19,20] which include the belief that maternal health issues fall within the woman’s domain and the fear of social stigma; economic issues [6,8,11,14,18,19,20] such as the nature of men’s work and poor economic status; couple’s personal preferences, poor knowledge and attitudes [11,18,19]; and health systems barriers [8,11,14,18,20,21], for example, a lack of services targeting men, long queues, lack of privacy and space, as well as poor attitudes of healthcare workers. While studies have investigated challenges to MPI in maternal health, future research is recommended to strengthen the evidence base for this in different countries [8]. Within the South African context, there is limited research focusing on barriers to male engagement in maternal health care, with a 2024 scoping review of articles published between 2013 and 2023 identifying one study [8] and a 2023 systematic review and meta-synthesis referring to two studies [11] from South Africa.

Maternal health care is a free public health service in South Africa [1]. While private health facilities provide maternal health care services at a cost, the majority of the South African population (84%) are reliant on government health care provision [22]. The South African population is culturally and ethnically diverse, characterised by a wide range of languages, religious beliefs and customs [23], hence the recommendation to provide culturally appropriate health care [19,24,25,26] which is also enshrined in the Constitution of South Africa [27]. Therefore, this paper specifically focuses on the factors associated with limited MPI in maternal health care amongst Sesotho-speaking persons residing in the Mangaung Metropolitan Municipality in the Free State Province, South Africa.

## 2. Materials and Methods

### 2.1. Research Setting and Design

In terms of population size, the Free State is the second smallest of the nine provinces in South Africa, with a population of approximately 2.6 million. Although traditional family structures are evolving, they remain important given that large proportions of the population face severe poverty and unemployment, and institutional support is lacking. Of the households in the province, 44.6% are female-headed compared to 42.3% of households in the country; 23% of households in the Free State comprise a single person, generally limited to more affluent households, with most household compositions described as nuclear (43.8%) or extended families (31.6%). Approximately 42% of children live with their mothers (45.4% SA), 34.7% live with both parents (31.5% SA), 17.5% live with neither parent (19% SA), and 5.7% live only with their father (4.2% SA). In terms of social grants, the Free State has the second-highest percentage of households receiving social support (64% compared to 50% in SA). When compared to the other Metropolitan Municipalities in the country, individual (41.4%) and household (59.4%) grant receipt was the highest in Mangaung Metropolitan. Similarly, Mangaung was the metro where social grants were considered the main source of income, while salaries and wages were the least common. Food access problems were also most common in Mangaung (25.9% of households) [28]. It is estimated that 72.3% of the population in the Free State is Sesotho-speaking [29]. Maternal health care indicators for the metropolitan suggest that the metro is not fairing as well as the country in general. More specifically, ANC first visit coverage, which measures the proportion of pregnant women who attend at least one ANC visit, was 70.5% (the lowest in the province) compared to 75.6% in the country [30]. Earlier research among Sesotho-speaking women found that pregnancy disclosure was a sensitive issue, with women believing that secrecy was necessary to protect the unborn baby against witchcraft, a feared phenomenon in many African societies. Furthermore, pregnancy was seen as a normal transition period and not an illness; therefore, the women did not consider it necessary to commence early with ANC [31,32].

A mixed method, concurrent/parallel design [33] was applied to explore MPI in maternal health and to identify factors associated with limited MPI. A cross-sectional survey was undertaken among men with a biological child younger than six years of age to determine levels of participation in maternal health care as well as to identify factors associated with limited participation. Sixteen focus group discussions with men and women of reproductive age, as well as eight community discussions with community leaders, were also conducted to gain a more detailed understanding of the possible reasons for lower levels of MPI in maternal health. This design allowed the researchers to view the research problem from multiple perspectives and methods, and to triangulate and validate the data to enrich the findings of the study.

### 2.2. Sampling

The target population for the survey was Sesotho-speaking men residing in previous townships (i.e., underdeveloped residential areas reserved for non-whites during the Apartheid era, 1948–1994, which still exist today) of a metropolitan area in the Free State Province. A combination of purposive and convenience sampling was used to recruit 407 biological fathers (i.e., men who self-identify as being the natural or birth father of a child), 18 years and older, who had a biological child younger than six years of age. The target population for the focus group discussions were Sesotho-speaking men and women of reproductive age, specifically 18–49 years. The target population for the community discussions was community/traditional leaders, representatives of men’s groups, and traditional healers. All participants were recruited through non-governmental organisations, churches, and women’s and men’s forums, where posters and brochures advertising the project were available. Interested participants were invited to contact the fieldwork manager or to meet the fieldwork team at a designated venue and time.

### 2.3. Data Collection

Experienced male fieldworkers, conversant in both Sesotho and English, obtained informed consent and administered the survey questionnaires, which were available in English and Sesotho. The questionnaire collected information on biographical characteristics, childhood experiences, health, family planning, ANC, delivery, and gender-equitable norms. The Gender Equitable Men (GEM) scale [34] was translated into Sesotho and used to measure gender norms. The questions were answered on a three-point scale—agree, partially agree, and disagree. Factor analysis reduced the scale to two sub-scales. The first sub-scale focused more generally on inequitable gender norms and comprised five items: (1) “Men are always ready to have sex”, (2) “Men need sex more than women do”, (3) “You don’t talk about sex, you just do it”, (4) “Women who carry condoms on them are easy”, and (5) “A woman should obey her husband in all things”, with a Cronbach’s alpha coefficient of 0.63. The second sub-scale focused on gender-based violence and comprised three items: (1) “There are times when a woman deserves to be beaten”, (2) “A woman should tolerate violence to keep her family together”, and (3) “It is alright for a man to beat his wife/partner if she is unfaithful”, with a Cronbach’s alpha coefficient of 0.84.

A total of sixteen focus group discussions, eight with men of reproductive age and eight with women of reproductive age, were conducted. In addition, eight community discussions were undertaken. Experienced male facilitators conducted the focus group discussions and community discussions with the men, and experienced female facilitators conducted the focus group discussions with the women. All focus group and community discussions were conducted in Sesotho and were audio-recorded with permission from the participants. The discussions were held in a place most convenient for the participants and were facilitated by a main facilitator and one assistant facilitator, who was responsible for note-taking.

### 2.4. Data Analysis

Quantitative data was analysed in IBM SPSS version 29 [35]. The data was described using frequency counts and percentages for categorical variables and means and standard deviations (SD) for continuous variables. While MPI in maternal health is a multi-faceted concept, researchers tend to focus on a single aspect of involvement [36]. With this context in mind, the current study, in line with Galle et al.’s [7] male involvement framework, and previous South African research [9,10], identified eight potential questions to measure MPI in maternal health (response categories were Yes = 0 and No = 1).

- Communication: (1) Did you ever discuss family planning with the mother of your youngest child?
- Decision making: (2) Did you and the mother of your youngest child decide together how many children you would like to have?
- Practical support: (3) Did you provide the mother of your youngest child with financial support during her pregnancy? (4) Did you help the mother of your youngest child with household chores during her pregnancy?
- Physical support: (5) When the mother of your youngest child was pregnant, were you present at any of the ANC visits?
- Emotional support: (6) Did you provide the mother of your youngest child with emotional support during her pregnancy? (7) Did you encourage the mother of your youngest child to go for ANC visits?

Exploratory factor analysis reduced the number of items to seven (item 6, “Were you present in the delivery room at the birth of your youngest child?” was dropped as it did not load highly on any of the factors) and identified two distinct factors. Factor 1 related to support and decision-making (items 1, 2, 3, 4, and 7), while Factor 2 was focused specifically on ANC in terms of physical presence and encouragement (items 5 and 8). Less random and systematic errors exist when the total score of a scale is used; therefore, for this paper, the multiple linear regression analysis was conducted using all seven questions as a summated scale [37].

Newly developed scales tend to have lower alpha coefficients as they require further refinement. Reliability coefficients less than 0.60 are low, suggesting that the scale has limited internal consistency. The Kuder–Richardson (KR20) test is used to determine the internal consistency of a scale comprising of dichotomous variables [37], and revealed a coefficient of 0.603, which is above the 0.60 cut-off for internal consistency.

Multiple linear regression, with bootstrapping (1000 samples), was run to predict the factors associated with lower MPI (based on all seven items) from age, employment status, living with the mother of your youngest child, having a male role model engaged in household chores while growing up, challenges in accessing health services, men attending clinics are seen to be HIV positive, willingness to attend a maternal health care programme in future and gender inequitable norms.

The qualitative data from the focus group and community discussions were thematically analysed using an inductive approach [38]. The audio recordings were transcribed, and the transcriptions were translated into English. The transcriptions were read and reread for the purpose of familiarisation with the data. NVIVO 14 was used to sort and organise the data. Initial codes were generated by the second author, and similar codes were grouped together into categories. The codes and categories were refined, and themes were identified. To ensure the trustworthiness of the data, the criteria of credibility, transferability, dependability, and confirmability were observed [39]. Credibility was ensured through triangulating the data from different sources (i.e., biological fathers, men and women of reproductive age, and community leaders) as well as through the use of different research methods (i.e., a survey, focus group discussions, and community discussions).

## 3. Results

### 3.1. Biographic and Background Information

The average age of the participants was 33.11 years (SD 7.541). Slightly less than two-thirds of the men (*n* = 256; 63.2%) were unemployed. Almost three-quarters (*n* = 278; 73.7%) were living with the mother of their youngest child, and 80.2% (*n* = 325) had been living with their youngest child since birth (Table 1).

### 3.2. Experiences of the Public Health System

Slightly more than two-thirds of the survey participants (*n* = 255, 68%) had not experienced challenges in accessing health services at public clinics compared to 32% (*n* = 120) who had. Of the 120 men who experienced challenges in accessing healthcare, three quarters (*n* = 90, 75.8%) cited long waiting times, and 57.5% (*n* = 69) indicated poor attitudes of healthcare workers. More than a third of the participants (*n* = 141, 37.5%) believed that people assumed any man attending a public clinic would be HIV positive.

A focus group discussion with men residing in a small town suggested that they regarded public clinics as women’s spaces and only visited a clinic when faced with an emergency.

*Just like he has explained, there are people that believe that a man should not go to the clinic. Men do not attend clinics; they have seen this from old men that they do not go to the clinics. Even when we were children, we used to go with our mothers right, now our children go with their mothers. In other words that thing is not usual for a man to always go to the clinic. I think it is like that even today. A man will go to the clinic only when he goes to extract a tooth and he wants to be helped. Even when he goes to extract it is already bad (Yes). It is bad*.

(FGD4MenDewetsdorp)

### 3.3. Gender Roles and Norms

Almost two-thirds of the participants (*n* = 268, 65.8%) indicated that they had seen their father or other male figure involved with domestic chores. The focus group discussions with men and the community discussions with community leaders further elaborated on this:

*For a woman there is a certain manner that I want her to give me support. I want that when I come from work, she must put on hot water (Laughs) and pour it in a washbasin. When I eat, I don’t eat cold food I eat hot food. I don’t eat warmed food, only fresh from the pot meals. My house must be clean, and my clothes must be clean and ironed. Even when I have not bathed, I must look like that man lives with his woman. And the last one she must always have sex with me every day*.

(FGD4MenDewetsdorp)

*Many things are done by fathers, buying a house, providing food for the family. There are many things that involve the father. Now the question is, do we say fathers should live as fathers and again as mothers, meaning they must live a double life? They must do their duties as providers and women’s duties.*

(CommunityLeaders3Bloemfontein)

The mean score for the GEM sub-scale measuring inequitable norms related to sexual behaviours and obedience was 7.43 (SD 2.706), which falls in the middle of the range of possible scores (5–15). The mean score for the GEM sub-scale focused on inequitable norms and gender-based violence was 3.28 (SD 0.989), which was closer to the minimum score of 3 (range 3–9).

### 3.4. Male Partner Involvement in Maternal Health

The average score on the MPI scale was 1.3 (SD 1.428) and ranged from 0 to 6, with a higher score indicative of less MPI. Three out of five participants (63.9%) scored above the mean for higher levels of MPI in maternal health. With regard to the specific items on the scale, MPI was slightly lower on deciding how many children to have (28.4% no joint decision-making), discussions about family planning (28% no discussions), and helping with household chores during pregnancy (23.3% no assistance) (Table 2).

Focus group discussions with women of reproductive age supported this expanded concept of MPI. Some women referred to the importance of men being present at ANC visits.

*A father is mostly needed when a mother is pregnant. He must accompany her to the clinic although he won’t be able to stand by the queue. His presence puts a mother at ease. This is why we must always teach fathers to take responsibility even before a child is born. This thing of them taking responsibility only when the child is born, it is very wrong because they are like kids who don’t easily adapt. So, we must teach them.*

(FGD2WomenBloemfontein)

It is important to note that not all women wanted their partner to accompany them to the clinic for ANC.

*From my perspective, although most people appreciate it, I don’t like it when fathers go with mothers to the clinic. This is because pregnant mothers share sensitive and uncomfortable stories when they are together at the clinic. Imagine how your partner would feel when hearing them. For instance, you find that it is painful for other mothers to see supportive fathers at the clinic because they wish they had that support but they don’t. Hence, I would just suggest that my partner just accompanies me then leave or he might as well just come fetch me when I am done.*

(FGD2WomenBloemfontein)

Men described their attendance at ANC in terms of supporting their partner to go for check-ups and not necessarily physically going into the ANC room with her. Some men indicated walking with their partner to the clinic and waiting for her to finish there, while other men referred to taking an interest in what happened at the ANC visit and making physical arrangements for the delivery.

*Yes, for me sir to accompany the lady and take her to the clinic, she sometimes gets swollen feet when she is pregnant. Yes, I have to walk with her slowly until she arrives at the clinic then get her inside, then wait for her until she finishes at the clinic then take her back and see that she is comfortable.*

(FGD7MenBotshabelo)

*When the mother is pregnant, she goes for check-ups. Like right now my wife is pregnant (Yes), she can give birth any time next week. So, I support her every time when she goes for check-ups. When she comes back, we sit down and check her file on how she went, how much the baby weigh and then how is the baby’s heartbeat, things like that. I will even check the next date. On the date she should go there, she won’t even go there. I’m the one that goes to the clinic to get a transport form. I will come back and say mommy here is your transport form for this date.*

(FGD4MenDewetsdorp)

Feedback received from the women’s focus group discussions supported this, with the women indicating that their partners kept them a place in the queue at the clinic thereby allowing them to rest until it was their turn to see the nurse.

*I have realised that pregnant women stand for long hours on their feet at hospitals, so that is tiring for them. If their partners are there, at least she can sit down, and he will take over the queue for her.*

(FGD2WomenBloemfontein)

Women also referred to MPI in terms of support with household chores, such as lifting heavy objects, cooking, washing and collecting water. Furthermore, the importance of emotional support was also mentioned.

*He must cook, clean up and wash dishes because when I am pregnant, I can’t be able to carry a bucket full of water and bend. He should carry that bucket of water, and I should do only light things only like sweeping.*

(FGD6WomenThabaNchu)

*The father must stay close to the mother while still pregnant like when a person is pregnant they cry about many things, things like dizziness there are pains that appear out of nowhere and feet get swollen and is unable to do some of the things. So the father must always be there so that when the mother cries about something, he is there to assist quickly so that the mother doesn’t hurt herself.*

(FGD1WomenBloemfontein)

In addition to physical and emotional support, the women also noted that their partners need to provide financial support during pregnancy and after birth.

*I think he must contribute financially. Pregnant women must eat healthy so that the baby is born healthy as well. He must also buy the mother necessary clothes seasonally. The same must also apply towards his child.*

(FGD4WomenDewertsdorp)

*When he is out, I need to have money in case of emergency and he must provide that, so that I can be assisted even in his absence. If anything happens, I will be covered I will be able to be taken to hospital.*

(TraditionalHealers1Botshabelo-Female)

### 3.5. Factors Associated with Lower Levels of Male Partner Involvement in Maternal Health

Multiple linear regression (Table 3) was run to predict the factors associated with lower MPI from age, employment status (0 = employed, 1 = unemployed), living with the mother of your youngest child (0 = no, 1 = yes), having a male role model engaged in household chores while growing up (0 = no, 1 = yes), challenges in accessing health services (0 = no, 1 = yes), men attending clinics are seen to be HIV positive (0 = disagree, 1 = agree), willingness to attend a maternal health care programme in future (0 = yes, 1 = no) and gender inequitable norms related to domestic violence (a composite score was calculated from three items: (1) “There are times when a woman deserves to be beaten”; (2) “A woman should tolerate violence to keep her family together”; (3) “It is alright for a man to beat his wife/partner if she is unfaithful”). The assumptions of linearity, independence of errors, homoscedasticity, unusual points, and normality of residuals were met. Bootstrapping was used to help mitigate some of the biases associated with convenience sampling by providing more robust estimates of standard errors and confidence intervals [40]. The independent variables combined statistically significantly predicted the dependent variable, lower MPI, and explained 31.8% of the variance in MPI, F (8.37) = 21.151, *p* < 0.001, adjusted R2 = 0.318). Age (t = −3.801; *p* < 0.001), employment (t = 3.078; *p* = 0.005), living with mother of your youngest child (t = 6.465; *p* < 0.001), male role model involved with domestic chores (t = 3.590; *p* = 0.003), and willing to attend a maternal health program in future (t = 3.124; *p* = 0.005) made a statistically significant contribution to the prediction of lower MPI.

For every one-unit increase in age, there was a 0.034 decrease in MPI scores, which implies that older men were more likely than their younger counterparts to be engaged in maternal health. Compared to employed men, unemployed men were 0.4 times more likely to report lower MPI. Men who were not living with the mother of their youngest child were 1.016 times more likely than their counterparts to have lower involvement in maternal health. Compared to men who had a male role model involved in domestic chores, men who did not were 0.497 times more likely to be less involved in maternal health. Men who were not willing to attend future maternal healthcare programmes were 0.575 times more likely than their counterparts not to be involved in maternal health.

The focus group and community discussions provided a more in-depth understanding of the factors associated with limited MPI in maternal health, as identified in the multiple linear regression. With reference to unemployed men being less likely to accompany their partner to the clinic, female focus group participants noted that there was not always sufficient money for transport for their partner to also visit the clinic.

*Regarding wealth, sometimes you find that the two parents do not have enough money for transport when you are supposed to go to the clinic. That is where one must compromise and stay behind even if he wanted to come along.*

(FGD3WomenDewetsdorp)

Participants described relationship problems, conflicts within families, and unplanned pregnancies as reasons why men did not live with the mother of their child and how this impacted their involvement in maternal health.

*The barrier that I see is that the mother is now pregnant and now there are processes. We have to go to hospitals but there are conflicts. She no longer wants me to be there because now she is emotionally unattached to me. Then that becomes a barrier of not knowing how to help or what should I do to help this person. She does as she pleases, when she goes to the clinic she goes alone, when she is going to deliver the baby, she goes alone.*

(CommunityLeaders4Bloemfontein)

*As men the kind of challenges that we come across sir, when we come to the point of here is the lady, she is pregnant, I have to give her a full support. I cannot give her the full support because maybe at her house they do not like me. Er when I want to or try to give her support about this thing there are people who keep telling her that this person is doing this and that.*

(FGD7MenBotshabelo)

*Another contributing factor is parents who want to choose partners for their children. Some would hate your pregnant partner because she is from a disadvantaged family or background. They want to choose for you who to have kids with, forgetting that you love that other one they hate. For some men, they see it as an excuse to leave when you are pregnant and run away from their responsibilities although we both conceived that child together without our parents involved.*

(CommuntyLeaders7Dewetsdorp)

Discussions with male community leaders provided insights as to the influence of fathers or other significant male figures on MPI in household chores and maternal health care.

*It’s because me growing up, it comes from my father’s side. The way my father grew up is the way I am going to grow up. We were even taken away from women. We don’t even know how to take care of a woman at home because there are people talking like my brother has explained that people would say you are not supposed to do all those things for a pregnant woman, they are done by grannies.*

(CommunityLeaders8Dewetsdorp)

*I just wanted to explain that fathers should not play mothers’ role. Over and above our upbringing, the way we were raised is the problem. When we were growing up, we were told that you are a boy, this is your work or role, you are a girl this is your role. It is said you should strike the harmer whilst the iron is hot because when it has cooled down, it’s very hard. I grew up knowing that the broom is not mine, washing dishes is not my duty. My job was to work in the garden with a rake and water the garden. Everything starts there, our issue starts there.*

(CommunityLeaders3Bloemfontein)

These challenges were reiterated in the focus groups with women, where it was explained that men did not learn during childhood that they should participate in domestic work at home.

*There are such issues because some men when required to help, he will tell you that he is not supposed to do that. Another one will tell you that he never saw his father doing that, so he also won’t.*

(FGD3WomenDewetsdorp)

Men who were not willing to attend future programmes related to maternal health were also less likely to have been actively involved in maternal health care. Male focus group participants explained that they did not believe men should be participating in maternal health matters.

*Men should not be working with them because other women do not feel comfortable, they are not happy with it. I’m talking about laws that needs to change. You see I’m a human being, I have rights to ask that the law be changed. It should not be male doctors but female doctors that delivers the babies. Men should not be in there.*

(FGD4MenDewetsdorp)

*I’m saying most men that are here in the location (Township) don’t go to the clinics with their partners during pregnancy. It is well known only that one who is jealous will go there (All laugh). I have never heard a man say he is accompanying his partner to deliver; I have never seen him in God’s honest truth.*

(FGD4MenDewetsdorp)

While the multiple regression analysis did not identify access to healthcare facilities as a hinderance to MPI, these concerns were raised in the focus group discussions, particularly the lack of space for men to be present in consulting rooms and the attitudes of nurses.

*I just want a change in our clinics, the way our clinics operate lately. When you go to a clinic with your husband when you are pregnant, they tell us that the father of the child should wait outside there is no space in the clinic. That’s one of the things we experience at our clinics, we don’t know whether the nurses are jealous of us or what hahahaha, because that person is there to support me so why do they say he should stay outside when they are busy examining me? That is why men are so reluctant because there is no use for them to accompany us, he will know nothing about your pregnancy, you will be alone inside.*

(FGD7WomenBotshabelo)

Both female and male focus group participants also raised the concern that clinic nurses forced men to be tested for HIV when their partners were pregnant.

*For men it is a problem, it’s a huge problem. When we go to the clinic, we are being tested and all that stuff, you have to be with your partner. Men have this tendency when I tell him let’s go to the clinic, I tested so they told me I should come along with you, men even if you force them to go and test, they will never do it. Whether your results come back negative or positive. They hate testing and they hate clinics.*

(FGD5WomenThabaNchu)

*Yes sir, you have to because when the lady goes to the clinic when she arrives there at the clinic then it is said that the father is also wanted. They must enter in there so that they can get tested to see that the baby won’t have any illnesses.*

(FGD7MenBotshabelo)

In addition to the factors identified by the multiple regression to be associated with lower levels of MPI in maternal health, the focus group and community discussions also identified additional challenges. Some male participants, during the focus group discussions, were of the opinion that only jealous men accompanied their wives to ANC visits. Furthermore, infidelity also prohibited men from attending ANC.

*I don’t want to lie, I have lived near the clinic for over six years and most of the men that go to the clinic according to my observations, the ones that accompany their partners, it’s the jealous fathers (Laughs). Because the thing I will tell you even right now, the ones sitting in here, I have never even seen anyone of them there (Laughs). Passing by there with their pregnant partners, nope. I’m saying most men that are here in the location (Township) don’t go to the clinics with their partners during pregnancy. It is well known only that one who is jealous will go there (All laugh).*

(FGD4MenDewetsdorp)

*Most of us are not married, now you see. You have impregnated your fourth girlfriend. On the way to the clinic, you pass by three girlfriends (all laugh) two of them are the ones you love. Therefore, we as men we have our mistakes which makes us run away from such things like going with your girlfriend to clinic. If you are married, things become easier.*

(FGD1MenBloemfontein)

The female participants explained how alcohol abuse and use deterred MPI and prohibited men from attending clinic visits.

*If a person knows that you are going to the clinic on Monday, on the weekend he will be drinking nonstop. On Monday morning when you say let’s wake up and go to the clinic, he will say he is tired or he will leave you on the bed and go back to that place to remove hangover, even alcohol will disturb them from accompanying you. Alcohol is number yoh!*

(FGD1WomenBloemfontein)

*I go back again, men are troublemakers even now in here we are only women there are no men right? And where are they, they are still here in the location, there are many of them and they don’t even work, when you call them to come are they gonna come? They fail to support us as their wives during pregnancy so to you guys do you think they will come? They will just talk many things. They will just come only if you say I bought you a case of alcohol; men please come that is when you will see them.*

(FGD5WomenThabaNchu)

## 4. Discussion

A 2011 qualitative study in a large township in KwaZulu Nata, South Africa, exploring the meaning of MPI during pregnancy and newborn care within the HIV context, argued for a comprehensive and holistic definition of MPI in maternal and newborn health [41]. The argument for an expanded definition of MPI is evident in findings from a randomised control trial in Soweto, South Africa, that measured MPI based on attendance at ANC. An association was found between MPI and maternal mental health but not between MPI and exclusive breastfeeding and low birthweight [15]. This highlights the need for a more comprehensive definition of MPI, which is more representative of the diverse socio-economic and cultural contexts of the Sesotho-speaking communities in the Free State. In the absence of a context-specific definition of MPI in maternal health, we used Galle et al.’s [7] framework for male involvement, together with previous South African research [9,10], and measured MPI involvement in terms of the following: couple communication about family planning; decision-making concerning the desired number of children; practical support, including financial support and assistance with household chores; physical support via attending ANC with their pregnant partner; and finally emotional support including encouraging the pregnant partner to attend ANC. Research exploring the meaning and understanding of MPI in pregnancy-related care among men in rural South Africa [9] noted that while MPI is commonly defined as men attending ANC, few men in the rural communities of Mpumalanga Province, South Africa do this. The findings further highlighted that MPI was generally understood as supporting pregnant partners by providing financial and emotional support, running errands, and sharing household chores. A further form of support was reported as accompanying pregnant partners to ANC visits at the clinics; however, they did not necessarily go into the consultation room, rather keeping them a place in the queue or arranging transport. Nesane et al. [19] confirmed this in their study in Limpopo, South Africa, where men transported their pregnant partners to the clinic for ANC but did not go inside the clinic. Our study also identified that in addition to their physical presence in the ANC room, men included walking with their partner to the clinic, waiting outside the clinic until their partner had seen the nurse, asking about the visit, and booking transport for the delivery, as some of the ways that they attended to ANC. Not all female respondents supported the idea of their partners’ presence in the clinic and ANC consultation room, as this could hinder them from sharing sensitive stories with other pregnant women and could also make women without their partners feel unsupported. Women did, however, support the inclusion of emotional and financial support as well as assistance with household chores in MPI during pregnancy.

Overall, we found higher levels of MPI in maternal and neonatal health, with the survey data identifying that age, employment status, living with the mother, having a male role model involved with domestic chores, and a willingness to attend future maternal health programs as factors predicting MPI. More specifically, younger men, unemployed men, men not living with the mother of their youngest child, men who had not had a male role model involved in domestic chores, and men who were not interested in attending future maternal health programmes were inclined to be less involved in maternal health. The qualitative data supported these findings and also highlighted that health system challenges and alcohol abuse prevented MPI in maternal health. Although there is limited research in South Africa on MPI in maternal health, we have identified some similar findings. Nesane et al. [16], in their study in Limpopo Province, also identified that unemployed men provided their pregnant partners with money for transport to the clinic, opting not to accompany them to save costs. An earlier study in KwaZulu Natal found that employed men could not attend counselling sessions during an MPI intervention because employment schedules conflicted with clinic hours, as well as the inability to obtain permission from employers for purposes of attending ANC [42]. There is a possibility that this has changed over the years as policies on family responsibility/ paternity leave have evolved since 2005 in South Africa. A study in a rural district in Mpumalanga Province investigating MPI in the prevention of mother-to child transmission of HIV found that men living with their partner were more likely to be involved in certain activities [10]. This supports our finding that men not living with the mother of their youngest child were less involved in maternal health.

Cultural barriers and gender roles have been identified as obstacles to MPI [19] where maternal health was viewed as women’s responsibility, and men were not allowed to take part in ANC. While our study did not specifically refer to these cultural practices, we found that the lack of a male role model while growing up who assisted with housework and caring for children was also associated with less MPI in maternal health. Findings from our focus group discussions further illustrated that stereotypical gender roles are still considered to be important by men, e.g., “fathers should not play mother’s role”, who expected women to take care of the household tasks and that women, especially the grannies, should support each other during pregnancy. Given the limited research in South Africa, we looked at findings from other African countries that also identified gender roles as preventing men from supporting their pregnant partners with domestic work [18,43], with men considering maternal health to fall within the domain of women’s responsibilities [16,17,20,43,44]. Furthermore, it was found that men who visibly supported a pregnant partner would be taunted/feared being taunted by male friends [17,45]. Likewise, men in our study noted that only jealous men accompanied their partners to ANC, which was reported with much laughter during the discussions.

While there was not a significant association between experiencing challenges in accessing health facilities and MPI in maternal health, almost a third of our participants experienced difficulties, particularly long waiting times and negative attitudes of healthcare workers. Furthermore, the qualitative findings also highlighted that the lack of space for men to be present in consulting rooms and the attitudes of nurses made it difficult for them to be present during ANC visits. A lack of space at clinics and hospitals was identified as a challenge to MPI in other studies conducted in South Africa, particularly in the Limpopo Province [16] and rural areas of Mpumalanga [10], where it was also reported that negative attitudes of healthcare workers deterred MPI in maternal health. These health systems challenges may have negatively influenced participants who stated that they were not willing to attend any future programmes focused on maternal health. Participants who would not participate in future programmes were also more likely not to have been involved in maternal health care.

The strengths of this study include using multiple items to measure MPI, as well as the mixed methods approach to collect and analyse data, which allowed for a more comprehensive understanding of the reasons for a lack of MPI in maternal health. Furthermore, the inclusion of a variety of Sesotho-speaking community members provided a more holistic description of MPI. However, as with all research, this study has limitations. The cross-sectional nature of the study did not allow for the interpretation of causality. We attempted to address this by triangulating the qualitative data from the focus group and community discussions with the quantitative survey data to gain more understanding of the barriers to MPI in maternal health. In addition, convenience sampling does not allow for the findings to be generalized. To enhance the robustness of our findings, we applied bootstrapping during the multiple linear regression analysis. This approach helps to mitigate some of the biases inherent in convenience sampling by providing more reliable estimates of standard errors and confidence intervals, thereby strengthening the validity of our results.

Since the questionnaires were administered by fieldworkers, response bias might have occurred, with participants potentially inclined to provide more favourable responses. This could partly account for, among others, the high levels of self-reported participation in family planning communication and ANC attendance. Future research should further examine the validity and reliability of measures of MPI in maternal health. Given the exploratory nature of this study, which contributes to the limited body of research on MPI in maternal health in South Africa, future research should undertake more robust methods such as random sampling and include a broader definition of MPI for a more holistic perspective of the barriers. For example, there should be separate questions asking about men’s presence in the ANC consulting room versus men who accompany their pregnant partners to the clinic but who do not necessarily go inside the clinic.

Furthermore, while previous research on ANC and Sesotho women highlights the involvement of family members such as mothers, mothers-in-law, and grandmothers during pregnancy [32], we can assume that pregnant women will, in most cases, be supported and not be residing alone. Approximately 23% of households in the Free State comprise a single person, generally limited to more affluent households, with most household compositions described as nuclear (43.8%) or extended families (31.6%) [28]. Nevertheless, future research should also investigate who, in addition to her partner, supports her during her pregnancy and what the nature of that support is.

While this study focused on the factors associated with lower levels of MPI in maternal health, it was evident that many men are involved partners and are willing to be involved. Therefore, it is important for healthcare facilities providing ANC and non-government organisations working in the field of maternal health to take cognisance of the factors that hinder men from becoming more involved. In this regard, interventions aimed at encouraging MPI should approach male involvement from the needs of women and men, i.e., a gender-transformative approach should be followed. In a gender-transformative approach, men and women work together to identify gender inequitable norms that act as barriers to sexual and reproductive health as well as maternal and child health services and then to develop practical solutions [46,47]. Within this context, it is equally important to consider cultural nuances that either hinder or enable men to be involved partners in maternal health. Transformation is also required at the health systems level to facilitate a gender-transformative, culturally sensitive approach to MPI in maternal health.

## 5. Conclusions

Although MPI in maternal health is recognised as one of many strategies that can work towards the improvement of maternal morbidity and mortality outcomes, it remains limited in many regions worldwide, not least of all in South Africa. In this study, socio-demographic (not living with the mother of the child and employment status), cultural (gender norms), and health systems (not wanting to participate in maternal health interventions) factors contributed to a lack of MPI in maternal health. Health systems capacity building (e.g., through the use of community health workers) is required for the promotion of male partner interventions that are in line with gender-transformative approaches as well as socio-cultural practices and gender norms.

## Figures and Tables

**Table 1:** Biographic information.

Characteristics	N	%
Mean age	Mean 33.11 SD 7.541	
Highest level of education	N = 404	%
Primary (Grades 1–7)	17	4.2
Secondary (Grades 8–12)	339	83.9
Tertiary	48	11.9
Employment status	N = 405	
Employed	149	36.8
Unemployed	256	63.2
Receiving social grants	N = 402	%
Yes	163	40.5
No	239	59.5
Current marital status	N = 405	%
Married	89	22.0
Not married but in a relationship	290	71.6
Not married and not in a relationship	26	6.4
Living with the mother of your youngest child	N = 377	%
Yes	278	73.7
No	99	26.3
Youngest child has lived with you since birth	N = 405	%
Yes	325	80.2
No	80	19.8

**Table 2:** Male partner involvement in maternal health.

Item	N	%
Discussed family planning (N = 405)		
Yes	293	72.0
No	114	28.0
Decision made on number of children to have (N = 405)		
Yes	290	71.6
No	115	28.4
Provide financial support during pregnancy(N = 407)		
Yes	377	92.6
No	30	7.4
Provide support with household chores during pregnancy (N = 407)		
Yes	312	76.7
No	95	23.3
Provide emotional support during pregnancy (N = 407)		
Yes	346	85.0
No	61	15.0
Present at ANC visits		
Yes	335	82.3
No	72	17.7
Encourage pregnant partner to attend ANC		
Yes	381	93.6
No	26	6.4

**Table 3:** Determinants of lower male partner involvement in maternal health care (seven-item scale).

Variables	B	Standard Error *	β	t	*p*-Value *	BCa 95% CI *
Age	−0.034	0.008	−0.182	−3.801	<0.001	−0.049–0.017
Employment	0.400	0.123	0.140	3.078	0.005	0.176–0.640
- Employed (ref)						
- Unemployed						
Living with mother of youngest child	1.016	0.173	−0.323	−6.465	<0.001	0.686–1.334
- Yes (ref)						
- No						
As a teenager observing male figure involved with domestic chores	0.497	0.151	−0.168	−3.590	0.003	0.232–0.731
- Yes (ref)						
- No						
Challenges in accessing healthcare facilities	0.043	0.140	0.014	0.304	0.766	−0.212–0.317
- No (ref)						
- Yes						
Men attending clinics considered to be HIV+	0.058	0.125	0.020	0.449	0.654	−0.174–0.310
- Disagree (ref)						
- Agree						
Willing to attend maternal health programme	0.575	0.194	0.152	3.124	0.005	0.184–0.974
- Yes (ref)						
- No						
GEM domestic violence	0.054	0.074	0.038	0.845	0.475	−0.078–0.227

* Bootstrap results based on 1000 bootstrap samples.

## Data Availability

The raw data supporting the conclusions of this article will be made available by the authors upon request.
